# Lower age at menarche affects survival in older Australian women: results from the Australian Longitudinal Study of Ageing

**DOI:** 10.1186/1471-2458-10-341

**Published:** 2010-06-15

**Authors:** Lynne C Giles, Gary FV Glonek, Vivienne M Moore, Michael J Davies, Mary A Luszcz

**Affiliations:** 1 Life course and Intergenerational Health Research Group, Robinson Institute, The University of Adelaide, Adelaide South Australia 5005, Australia; 2 Discipline of Public Health, The University of Adelaide, Adelaide South Australia 5005, Australia; 3 Discipline of Statistics, The University of Adelaide, Adelaide South Australia 5005, Australia; 4 Discipline of Obstetrics and Gynaecology, Robinson Institute, The University of Adelaide, Adelaide South Australia 5005, Australia; 5 School of Psychology and Flinders Centre for Ageing Studies, Flinders University, Adelaide South Australia 5001, Australia

## Abstract

**Background:**

While menarche indicates the beginning of a woman's reproductive life, relatively little is known about the association between age at menarche and subsequent morbidity and mortality. We aimed to examine the effect of lower age at menarche on all-cause mortality in older Australian women over 15 years of follow-up.

**Methods:**

Data were drawn from the Australian Longitudinal Study of Ageing (n = 1,031 women aged 65-103 years). We estimated the hazard ratio (HR) associated with lower age at menarche using Cox proportional hazards models, and adjusted for a broad range of reproductive, demographic, health and lifestyle covariates.

**Results:**

During the follow-up period, 673 women (65%) died (average 7.3 years (SD 4.1) of follow-up for decedents). Women with menses onset < 12 years of age (10.7%; n = 106) had an increased hazard of death over the follow-up period (adjusted HR 1.28; 95%CI 0.99-1.65) compared with women who began menstruating aged ≥ 12 years (89.3%; n = 883). However, when age at menarche was considered as a continuous variable, the adjusted HRs associated with the linear and quadratic terms for age at menarche were not statistically significant at a 5% level of significance (linear HR 0.76; 95%CI 0.56 - 1.04; quadratic HR 1.01; 95%CI 1.00-1.02).

**Conclusion:**

Women with lower age at menarche may have reduced survival into old age. These results lend support to the known associations between earlier menarche and risk of metabolic disease in early adulthood. Strategies to minimise earlier menarche, such as promoting healthy weights and minimising family dysfunction during childhood, may also have positive longer-term effects on survival in later life.

## Background

The timing and development of the reproductive system can be viewed as a continuum across the lifespan [1] in which there is an intimate association with underlying metabolic processes, reproductive function and, potentially, chronic disease risk. The onset of menarche is an important milestone in a woman's reproductive career, and appears to be meaningfully related to a range of emergent chronic disease risk factors, and subsequent morbidity and mortality in later-life.

An association between lower age at menarche - that is, < 12 years [2] - and an increased risk of uterine cancer [3] and breast cancer [4,5] is well established. One explanation for the latter is that earlier menarche alters patterns of adiposity, which in turn appear to be related to an increased risk of breast cancer [6]. There is also a relationship between earlier age at menarche and cardiovascular disease in adolescence [2]. Earlier age of menarche has been commonly associated with increased body mass index (BMI) in childhood and adolescence [7], which appears to partially reflect restricted growth in very early life [8] followed by rapid post-natal growth [9]. These adverse changes also track and amplify over time into adulthood, such that earlier menarche is associated with increased risk of developing the metabolic syndrome in adulthood [10]. There is conflicting evidence concerning the effects of earlier age at menarche on cardiovascular mortality [11-13].

Somewhat surprisingly, few authors have examined the relationship between age at menarche and all-cause mortality. Previous studies were limited by inadequate adjustment for confounding factors [14] or through samples that were not representative of the wider population [12]. Lakshman et al.'s recent large study [13] partly addresses these limitations, but did not include some potentially important covariates such as age at menopause.

Therefore, the aim of the present study was to examine the effect of earlier age at menarche on all-cause mortality over 15 years of follow-up in a sample of older women drawn from the general Australian population. We were also able to adjust for a broader range of reproductive, demographic, health, and lifestyle factors than in previous studies in this area.

## Methods

We drew data from the Australian Longitudinal Study of Ageing (ALSA) that began in 1992 in Adelaide, South Australia. ALSA's major objectives were to assess the effects of social, biomedical, behavioural, economic, and environmental factors upon age-related changes in the health and wellbeing of older persons. The study has been described in detail elsewhere [15,16]. Briefly, the primary sample was randomly selected from the South Australian Electoral Roll, and stratified by local government area, gender and age group (70 - 74, 75 - 79, 80 - 84 and > 85 years). Older males were over-sampled to ensure sufficient numbers for longitudinal follow-up. Persons were eligible for the study if they were resident in the Adelaide Statistical Division and aged 70 years or more on 31 December 1992 (the mid-date of the study's Wave 1). Spouses aged 65 years or more and other, non-spousal household members aged at least 70 were also invited to take part. A total of 1,031 of the 2,087 Wave 1 participants were female.

Ethical approval for the study was granted by the Flinders Committee for Clinical Investigation, and each participant gave written informed consent.

### Menstrual and reproductive variables

Wave 1 questions concerning menstrual and reproductive histories were used to derive variables reflecting these histories. Age at menarche (classified as < 12 or ≥ 12 years, after Remsberg et al. [2]), age at menopause (≤ 44, 45 - 49, 50 - 54, ≥ 55 years, or surgical), number of reproductive years (≤ 30, 31 - 39, and ≥ 40) and the number of live births (0, 1 - 2, 3 - 4, ≥ 5) were derived from the women's self-reported histories.

### Demographic, health and lifestyle variables

The analyses were adjusted for the effects of demographic, health, and lifestyle variables at study baseline demonstrated in previous studies to be associated with age at menarche or survival [12-14]. Demographic variables included age group and place of residence (community or residential care). Self-rated health was classified as excellent/very good, good, and fair/poor. The number of chronic conditions was derived from self-reported information on whether each participant had ever suffered from 10 common conditions, including diabetes, cancer and cardiovascular disease [13,17]. Cognitive function was dichotomised as impaired or intact based on results from a subset of items from the Mini-Mental State Examination [18,19]. Participants were classed as current, former, or never smokers from their responses to questions concerning smoking. Participants were classified as active or sedentary based on questions about the exercise undertaken in the fortnight prior to Wave 1 interview [16]. BMI was derived from height and weight measured at clinical assessment, and categorised as < 20, 20-25, and > 25 kg/m^2^.

### Statistical analyses

The bivariate associations between age group at menarche and the reproductive, demographic, health and lifestyle covariates were investigated through chi-square tests of association for categorical covariates and Mann-Whitney U-tests for covariates with a continuous distribution.

Survival status at the censoring date of 15 years after the Wave 1 interview was ascertained. The Epidemiology Branch of the Department of Health in South Australia conducted searches of official death certificates and deaths were confirmed by the South Australian Births, Deaths and Marriages bureau. Full name, date of birth and last known address of ALSA participants were used in the data linkage with the Deaths database. If no direct match was made, the Electoral Roll was checked for errors in birth dates, changes or errors in recorded name, and changes or errors in recorded address. Informants nominated by ALSA participants at Wave 1 were contacted if participants could not be located at subsequent interviews. The date of death supplied by informants was used if a participant died outside of South Australia. These methods of death ascertainment for ALSA participants have been validated previously [20,21].

The response variable was the number of days to death from the date of the Wave 1 interview for decedents and 5,497 days for participants who survived 15 years after their initial interview. Cox proportional hazards models were fit to the data to ascertain the hazard ratio (HR) for the effect of lower age at menarche on survival, controlling for the other demographic, health, lifestyle and reproductive characteristics. Models that sequentially adjusted for demographic, then health, then lifestyle, then reproductive characteristics were fit to investigate how these groups of variables modified the association, if any, between time to death and age at menarche. The Efron method was used to correct for ties in the time to death [22,23].

The fit of models was assessed using graphical methods based on martingale residuals [24,25]. The assumption of proportional hazards was assessed by regressing the scaled Schoenfeld residuals against the log of time and testing for zero slope [26]. Stata version 10.0 (College Station, Texas) was used in all analyses [27].

To assess the sensitivity of results to missing values for age at menarche and age at menopause, multiple imputation methods were used to estimate missing data for these two variables. Among other factors, nutrition, ethnicity and family composition are known to influence menses onset [28]. Therefore, in the multiple imputation analysis age at menarche (missing n = 42; 4%) was imputed 10 times according to the dependence of menarcheal age on number of sisters, BMI (adult BMI as a marker for childhood BMI), year of birth, and country of birth (Australia, England, other European country, other non-European country). Age at menopause (missing n = 77; 7%) was imputed 10 times according to its dependence on type of menopause (natural or otherwise), BMI, parity, age at first pregnancy, age at last pregnancy, number of breast-fed children, number of sisters, smoking status at time of menopause, age left full-time education, and country of birth. The 'ice' and 'micombine' packages in Stata were used in the imputations [29]. Complete case and imputed case analyses were run for all models and results are reported for both types of analyses.

## Results

The 1,031 women included in our analyses were aged between 65 and 103 at study baseline, with an average age of 77.3 years (SD 7.1). The mean age at menarche in these women was 13.6 years (SD 1.7), while the median was 14 years (interquartile range (IQR) 12 - 15 years). A total of 10.7% of the women experienced menarche aged less than 12 years, while 12.5% of the women were aged 16 years or more when their menses commenced. The average age of menopause among the women in our study was 48.1 years (SD 5.8; median 50 years, IQR 45 - 52). More than one fifth of the sample experienced menopause before 45 years of age, while 10.5% were aged 55 years or more when they experienced menopause. Three quarters of the women underwent natural menopause. The women in the study had an average of 34.5 reproductive years (SD 6.0; median 35; IQR 31 - 38). A total of 31 women (3%) reported they were using hormone therapy at the time of the Wave 1 interview.

Table 1 shows the reproductive, demographic, health and lifestyle characteristics and 15 year survival status of the female participants in Wave 1 of ALSA classified according to age at menarche. Of the 1,031 women who took part in Wave 1, 673 (65%) died within 15 years of their Wave 1 interview; the average length of follow-up for decedents was 7.3 (SD 4.1) years. Among the women with a reported age at menarche (n = 989), there were statistically significant associations between age at menarche and attained age group (X^2 ^= 9.73 on 4 df; P = 0.045), number of morbid conditions (Mann-Whitney U-test *z *= 2.51; P = 0.012), cognitive function (X^2 ^= 4.67 on 1 df; P = 0.031), BMI category (X^2 ^= 16.58 on 3 df; P = 0.001), and number of reproductive years (X^2 ^= 8.39 on 2 df; P = 0.015).

**Table 1:** Summary statistics for 1,031 female participants in wave 1 of ALSA classified according to age at menarche

		Age at menarche			
		
Characteristic	Overall	< 12	≥ 12	Missing	**P-value**^a^
	n = 1,031	n = 106	n = 883	n = 42	
Age group					
*65 - 69*	123 (12%)	14 (13%)	106 (12%)	3 (7%)	0.045
*70 - 74*	283 (27%)	41 (39%)	238 (27%)	4 (10%)	
*75 - 79*	241 (23%)	26 (25%)	211 (24%)	4 (10%)	
*80 - 84*	194 (19%)	14 (13%)	170 (19%)	10 (24%)	
*≥ 85*	190 (18%)	11 (10%)	158 (18%)	21 (50%)	
Place of residence					
*Community*	948 (92%)	100 (94%)	821 (93%)	27 (64%)	0.601
*Residential*	83 (8%)	6 (6%)	62 (7%)	15 (36%)	
Self rated health					
*Excellent/very good*	401 (39%)	32 (30%)	357 (40%)	12 (29%)	0.114
*Good*	324 (31%)	36 (34%)	269 (31%)	19 (45%)	
*Fair/poor*	306 (30%)	38 (36%)	257 (29%)	11 (26%)	
Number of morbid conditions					
*Median (IQR)*	3 (2 - 4)	3 (2 - 5)	3 (2 - 4)	2 (0 - 3)	0.012^d^
Cognitive function					
*Good*	888 (86%)	99 (93%)	758 (86%)	31 (74%)	0.031
*Poor*	143 (14%)	7 (7%)	125 (14%)	11 (26%)	
Smoking status					
*Never smoker*	718 (69%)	66 (62%)	618 (70%)	35 (83%)	0.266
*Former smoker*	229 (23%)	29 (28%)	193 (22%)	7 (16%)	
*Current smoker*	84 (8%)	11 (10%)	72 (8%)	1 (2%)	
Sedentary					
*No*	541 (52%)	54 (50%)	461 (52%)	26 (62%)	0.805
*Yes*	490 (48%)	52 (50%)	422 (48%)	16 (38%)	
Body Mass Index					
*< 20 kg/m^2^*	41 (4%)	2 (2%)	39 (4%)	0 (0%)	0.001
*20 - 25 kg/m^2^*	280 (27%)	18 (17%)	254 (29%)	8 (19%)	
*> 25 kg/m^2^*	428 (42%)	64 (60%)	355 (40%)	9 (21%)	
*Missing*	282 (27%)	22 (21%)	235 (27%)	25 (59%)	
Natural menopause					
*No*	273 (26%)	32 (30%)	219 (25%)	22 (52%)	0.229
*Yes*	758 (74%)	74 (70%)	664 (75%)	20 (48%)	
Age at menopause					
*≤ 44^b^*	104 (10%)	15 (14%)	89 (10%)	0 -	0.123
*45 - 49*	192 (19%)	19 (18%)	172 (19%)	1 (4%)	
*50 - 54*	316 (31%)	33 (31%)	276 (31%)	7 (27%)	
*≥ 55*	148 (15%)	7 (7%)	129 (15%)	12 (46%)	
*Surgical*	254 (25%)	32 (30%)	216 (24%)	6 (23%)	
Reproductive years					
*≤ 30^c^*	218 (23%)	20 (19%)	198 (24%)	-	0.015
*31 - 40*	608 (65%)	63 (60%)	545 (65%)	-	
*≥ 40*	116 (12%)	22 (21%)	94 (11%)	-	
Parity					
*0*	136 (13%)	14 (13%)	113 (13%)	9 (21%)	0.527
*1 - 2*	439 (43%)	44 (42%)	372 (42%)	23 (55%)	
*3 - 4*	351 (34%)	41 (39%)	302 (34%)	8 (19%)	
*≥ 5*	105 (10%)	7 (7%)	96 (11%)	2 (5%)	
Age first pregnancy					
*Median (IQR)*	25 (22 - 28)	24 (21 - 28)	25 (22 - 28)	26 (22 - 35)	0.149^d^
Age last pregnancy					
*Median (IQR)*	33 (29-37)	33 (29 - 36)	33 (29 - 37)	31 (29 - 36)	0.425^d^
Dead at 15 years follow-up					
*No*	358 (35%)	34 (32%)	319 (36%)	5 (12%)	0.411
*Yes*	673 (65%)	72 (68%)	564 (64%)	37 (88%)	

**Shown are n (%) unless otherwise noted**.

a: P-value excludes missing category in calculation

b: n = 954 available age at menopause

c: n = 942 available reproductive years

d: P-value based on Mann-Whitney U-test; all other shown P-values based on chi-square tests of association

Younger age group, living in the community, better self-rated health, fewer co-morbid conditions, better cognitive function, never smoking and exercise were jointly significant predictors of longer survival and subsequent analyses adjusted for these variables. We also controlled for BMI category, parity > 0, age at menopause, and number of reproductive years in the analyses, based on the significant effects of these variables on all-cause mortality reported previously [12,13]. The adjusted hazard ratios associated with each of these variables are presented in Table 2. The assumption of proportional hazards was tenable in the age-adjusted (global test X^2 ^= 3.26 on 5 df, P = 0.660) and all covariate adjusted (global test X^2 ^= 25.73 on 23 df; P = 0.314) analyses.

**Table 2:** Adjusted hazard ratios for effect of covariates on 15-year survival

Variable	HR^a^	95% CI^b^
Age group		
*65 - 69*	1.00	
*70 - 74*	1.62	1.10 - 2.38
*75 - 79*	3.43	2.35 - 5.00
*80 - 84*	5.73	3.86 - 8.50
≥ 85	9.08	6.02 - 13.70
Dwelling		
*Community*	1.00	
*Residential aged care*	1.91	1.41 - 2.57
Self rated health		
*Excellent/very good*	1.00	
*Good*	1.40	1.14 - 1.72
*Fair/poor*	1.42	1.15 - 1.77
Number of morbid conditions	1.06	1.01 - 1.11
Cognitive impairment		
*No*	1.00	
*Yes*	1.58	1.25 - 1.99
Smoking status		
*Never*	1.00	
*Former*	1.25	1.02 - 1.54
*Current*	2.02	1.50 - 2.71
Sedentary		
*No*	1.00	
*Yes*	1.31	1.10 - 1.56
Body Mass Index (kg/m^2^)		
*20 - 25*	1.00	
*< 20*	0.93	0.60 - 1.44
*> 25*	0.93	0.75 - 1.14
*Missing*	1.09	0.87 - 1.36
Parity		
*Nulliparous*	1.00	
> 0	0.87	0.69 - 1.09
Age at menopause		
*≤ 44*	1.01	0.72 - 1.43
*45 - 49*	1.10	0.83 - 1.40
*50 - 54*	1.00	
*≥ 55*	0.74	0.53 - 1.04
*Surgical*	0.98	0.76 - 1.27
Reproductive years		
*≤ 30*	1.00	
*31-39*	0.99	0.72 - 1.38
*≥40*	0.99	0.69 - 1.42

a: Hazard Ratios adjusted for other covariates

b: 95% confidence interval

As shown in Table 3, the effect of lower age at menarche overall was associated with approximately a one third increase in the hazard of death over a 15 year follow-up period (age adjusted HR 1.35; 95%CI 1.05 - 1.73). The effect persisted when adjusted for other covariates, although the hazard reduced to marginal statistical significance when other reproductive covariates were included in addition to health, lifestyle and demographic covariates (adjusted HR 1.28; 95%CI 0.99 - 1.65). Results did not differ substantively in the analyses based on the multiply imputed data (Table 3).

**Table 3:** Summary of effect of lower age at menarche on 15-year survival

	Complete case analyses (n = 942)			Multiple imputation analyses (n = 1,031)		
Model	HR	95% CI	P-value	HR	95% CI	P-value
1: Age adjusted						
*Menarche ≥ 12y*	1.00			1.00		
*Menarche < 12y*	1.35	1.05 - 1.73	0.018	1.30	1.02 - 1.65	0.032
						
2: Age + Health adjusted^a^						
*Menarche ≥ 12y*	1.00			1.00		
*Menarche < 12y*	1.31	1.02 - 1.68	0.037	1.26	0.98 - 1.61	0.070
						
3: Age + Health + Lifestyle adjusted^b^						
*Menarche ≥ 12y*	1.00			1.00		
*Menarche < 12y*	1.31	1.02 - 1.69	0.035	1.29	1.01 - 1.64	0.045
						
4: Age + Health + Lifestyle + Reproductive adjusted^c^						
*Menarche ≥ 12y*	1.00			1.00		
*Menarche < 12y*	1.28	0.99 - 1.65	0.062	1.25	0.98 - 1.60	0.074

a: Overall analyses adjusted for age group, place of residence, and health variables (self-rated health, cognitive function, number of morbid conditions).

b: Overall analyses adjusted for age group, place of residence, health variables, and lifestyle variables (smoking status, exercise status, BMI category).

c: Overall analyses adjusted for age group, place of residence, health variables, lifestyle variables, and reproductive variables (parity, age at menopause, number of reproductive years)

We repeated the analyses with age at menarche as a continuous variable and included a quadratic term. The results showed the adjusted HR corresponding to the linear term for age at menarche was 0.76 (95%CI 0.56 - 1.04; P = 0.091) and the adjusted HR corresponding to the quadratic term was 1.01 (95%CI 1.00-1.02: P = 0.083).

## Discussion

Younger age at menarche (i.e. < 12 years) appears to be associated with roughly a one third increased hazard of death over a 15 year period among Australian women aged 65 years or more, after adjustment for a broad range of reproductive, health and lifestyle variables. Sensitivity analyses using multiple imputation of missing data did not change the substantive conclusions. However, when considered as a continuous variable, age at menarche was not associated with an increased hazard of death at a conventional 5% level of significance. This suggests that any relationship between menarcheal age and mortality is not of a simple functional form.

The results from this study are also in broad agreement with the two recent studies by Jacobsen and colleagues [12,14], although there are some important differences between their work and the present study. In an earlier study, Jacobsen and colleagues had established a modest effect of age at menopause on all-cause mortality in the subset of naturally menopausal women within their Norwegian cohort [30]. However, they do not appear to have adjusted for age at menopause in their final analyses concerning the effect of age at menarche on all-cause mortality in the same cohort [14]. This may be a minor concern, as our findings concerning lower age at menarche did not differ substantively when age at menopause was excluded from the fitted model. More than one third of the women in the Californian cohort were pre-menopausal while almost half of the post-menopausal woman had a surgically-induced menopause [12]. In contrast, all of the women in the ALSA study had undergone menopause at least a decade prior to their Wave 1 interview, and less than one quarter of the women in our study had a surgical menopause. Our adjusted HR of 1.28 (95%CI 0.99 - 1.65) is also broadly consistent with that reported by Lakshman et al. (adjusted HR 1.22; 1.07-1.39). While the latter study was larger, with close to 16,000 women, the women in Lakshman et al.'s study were younger at baseline (range 40-79 years) and some remained pre-menopausal at follow-up. In contrast, women had to have reached age 65 to be eligible for inclusion in our study, so it is possible that left-censoring is in operation. Women with earlier menarche who died before the age of 65 could not enter our study, but this will not alter the effect of age at menarche on longevity among women who have already survived to older age. Thus we argue that our results more accurately represent the effect of age at menarche on mortality in a heterogeneous, post-menopausal sample of older women.

Menstrual onset follows from a cascade of endocrine changes that include increases in the secretion of gonadotropin-releasing hormone, growth hormone and insulin [6]. The activation of menarche is modulated by the hypothalamic-pituitary-gonadal system, particularly endogenous estradiol and lower sex hormone binding globulin [31,32]. While a broad range of genetic and environmental influences have been proposed to affect age at menarche [28], the pathways through which these influences operate remain poorly understood [33]. Genetic inheritance through the mother, growth and weight during infancy and early childhood [34,35], socioeconomic factors [36], and the quality of father-daughter relationships in early childhood [37] have all been highlighted as important factors that may serve to trigger menstrual onset. It is possible that the benefits from interventions that target menarche triggers will continue to accrue in older age, and further research effort in this area appears necessary.

There are several pathways posited through which age at menarche may impact on subsequent survival. Early menarche is associated with increased body fatness in adult women [10,38], which may in turn be associated with cardiovascular disease. Recent work has suggested that early menarche may not in itself be a determinant of an unfavourable cardiovascular profile, but may reflect negative metabolic imprinting during pre-pubescence [38]. Low birth weight and greater weight gain up to the age of 8 years have recently been demonstrated to predict younger age at menarche [39], so it may be that the effect of lower age at menarche on survival is reflecting events much earlier in the life course.

Early menarche has also been associated with an increased risk of breast cancer [4,5], and it is thought that the early exposure to the 'hormonal milieu' associated with regular menstrual cycles may be important in the aetiology of the disease. It is also possible that a lower age at menarche leads to a greater number of reproductive years, given that age at menarche and age at natural menopause are not highly correlated [40]. In turn, a greater number of reproductive years has been linked to a reduced lifespan [41]. An alternative view is that women who are biologically older than their chronological age (i.e. those women with a younger age at menarche) also die at a younger age than those women who experienced menarche aged 12 years or more.

The findings from the present study must be interpreted with several caveats. Although we adjusted for many reproductive, demographic, health and lifestyle covariates, complete data were unavailable for some potentially important factors such as use of oral contraceptives and lifetime use of hormone replacement therapy. However, oral contraceptives were not widely available during the cohort's reproductive lifetime, given that the youngest women in the study were aged 65 years in 1992 (and thus aged 34 years in 1961 when oral contraceptives first became available in Australia). Perhaps more serious is our lack of data concerning lifetime use of hormone replacement therapy. At the time of the baseline interview, three per cent of the women reported current hormone therapy use, but we do not know how many of the women in our study had previously used hormone therapy. However, the youngest women in our cohort were perimenopausal in the early 1980s, preceding the widespread use of hormone therapy. Therefore, we believe that the proportion of women in our cohort who had used hormone therapy in the past would be small and have minimal impact on our findings.

Another limitation in this study was that self-reported age at menarche was recalled a minimum of five decades after menses onset. The reliability of menarcheal age recalled in women aged 65 years or more does not appear to have been reported in the extant literature. Studies that have examined the reliability of age at menarche recalled in middle age have had mixed findings, with two studies reporting strong agreement [42,43]. A third study reported only moderate agreement of age at menarche collected in adolescence and again in middle age, although the authors suggested that categorising menarcheal age may improve the reliability of the measure [44]. Opportunities that may exist to examine the reliability of recalled age at menarche in cohorts of older women should be explored.

The average menarcheal age of 13.6 years reported in our study of older women was similar to the average age at menarche of Australian schoolgirls reported in 1932 (13.1 years; [45]) and 1948 (13.7 years; [46]). Given that the women in our study were born over a 38 year period between 1889 and 1927, it is possible that there was a secular trend towards a lower menarcheal age in the younger cohort members [47]. However, the association between age group and age at menarche was only weakly statistically significant in our study. This suggests that if such a trend existed in our cohort of women, its impact on the results is likely to be small. More broadly, our findings suggest that decreasing age at menarche could lead to shorter life spans. However, the societal secular trend of decreasing age at menarche has occurred concurrently with major advances in medical treatments and technologies that can lengthen life. Thus the apparent contradiction between decreasing age at menarche and increased longevity is plausible given the broader environment in which any change in age at menarche between subsequent generations has taken place.

Cause of death data are not yet available for the ALSA cohort at the 15 year follow-up, but future work is planned to examine the effect of age at menarche on cardiovascular mortality and deaths from breast cancer. It is possible that the relatively small sample size may lead to analyses with inadequate statistical power when separate causes of death are examined. Also noteworthy is that ALSA was not explicitly designed to examine the effects of reproductive factors on mortality, and the analyses reported here are based on self-reported data and adjust for covariates measured at baseline. However, these latter limitations are true of the majority of studies that have considered reproductive factors and survival. We believe these restrictions are balanced by ALSA's strengths, which include the richness of the baseline data, the Australian setting, and the inclusion of residents in aged care facilities. ALSA included a more heterogeneous population sample than many other longitudinal studies of ageing.

## Conclusions

In summary, our findings suggest that over a 15 year follow-up period, older women who began menstruating aged less than 12 years had an increased risk of overall mortality compared to women with a menarcheal age of 12 years or more.

## Competing interests

The authors declare that they have no competing interests.

## Authors' contributions

LCG participated in the design and conduct of the study, performed the statistical analyses and drafted the manuscript. GFVG performed the statistical analyses and helped to draft the manuscript. VMM and MJD participated in the design of the study and helped to draft the manuscript. MAL conceived of the ALSA study, and participated in its design and coordination and helped to draft the manuscript. All authors read and approved the final manuscript.

## Pre-publication history

The pre-publication history for this paper can be accessed here:

http://www.biomedcentral.com/1471-2458/10/341/prepub
